# 872. Assessing Racial and Geographic Disparities Across the Entirety of Burns Literature

**DOI:** 10.1093/jbcr/irag033.349

**Published:** 2026-04-06

**Authors:** Francesco M Egro, Kian Daneshi, Sarah M Tepe, Christopher J Fedor, Mare G Kaulakis

**Affiliations:** University of Pittsburgh Medical Center, Pittsburgh, PA; School of Population Health and Medicine, University of Sheffield, London, England; University of Pittsburgh Medical Center, Pittsburgh, PA; University of Pittsburgh Medical Center, Department of Plastic Surgery, Pittsburgh, PA; University of Pittsburgh Medical Center, Mercy Burn Center, Pittsburgh, PA

## Abstract

**Introduction:**

Geographic and ethnic diversity in medical research leadership remains poorly understood, with limited systematic analysis across medical specialties. This study presents the first comprehensive geographic and ethnicity analysis of first authorship patterns in burns literature, examining demographic disparities and temporal trends across four decades.

**Methods:**

In total 18 275 articles were analyzed from 12 major burns journals (1982-2025) using automated PubMed data mining. A novel multi-layer demographic inference system combining cross-reference resolution of author initials, comprehensive international surname databases, Unicode script detection, and fuzzy matching achieved 87.8% gender classification success (16 040 articles). For ethnicity classification, we implemented a comprehensive ensemble methodology incorporating web-enhanced surname databases (844 global surnames), advanced pattern recognition, and multiple confidence-tiered classification methods, achieving exceptional 73.2% classification success (11 742 articles) from the gender-classified subset.

**Results:**

Of 16 040 gender-classified articles, demographic inference successfully classified 11 742 authors (73.2% coverage) across major world populations. Asian authors comprised 43.5% of classified articles (5111), White authors 31.9% (3748), Middle Eastern 10.7% (1257), Black/African 8.7% (1024), and Hispanic/Latino 5.1% (602). Temporal analysis revealed statistically significant evolving patterns in international collaboration and geographic representation over four decades (χ^2^ = 119.9, *p*<.001), with Asian representation significantly increasing from 37.9% (1980s) to 47.2% (2020s) (*p*=.006), while maintaining substantial global diversity across all major ethnic groups and geographic regions.

**Conclusions:**

This study demonstrates comprehensive geographic and ethnic representation in burns literature authorship, with robust global diversity across all major population groups. Asian populations show strong representation (43.5%), with substantial White (31.9%), Middle Eastern (10.7%), Black/African (8.7%), and Hispanic/Latino (5.1%) contributions. These findings establish the burns field as internationally diverse while providing comprehensive benchmarks for global academic equity assessment and supporting evidence-based diversity initiatives.

**Applicability of Research to Practice:**

The findings of this project establish the field of burns as an inclusive and diverse specialty across ethnicities.

**Funding for the study:**

N/A.

